# Black tea aroma inhibited increase of salivary chromogranin-A after arithmetic tasks

**DOI:** 10.1186/s40101-018-0163-0

**Published:** 2018-01-24

**Authors:** Ai Yoto, Natsuki Fukui, Chisa Kaneda, Shoko Torita, Keiichi Goto, Fumio Nanjo, Hidehiko Yokogoshi

**Affiliations:** 1 School of Food and Nutritional Sciences, University of Shizuoka, Shizuoka, Japan; 2 College of Bioscience and Biotechnology, Chubu University, Kasugai, Japan; 3 Food Research Laboratories, Mitsui Norin Co., Ltd., Fujieda, Japan; 4 School of Marine Science and Technology, Tokai University, Shizuoka, Japan

**Keywords:** Black tea, Chromogranin-A, Acute stress, Profile of Mood States

## Abstract

**Background:**

Growing attention has been paid to the effects of food flavor components on alleviating negative brain functions caused by stressful lifestyles. In this study, we investigated the alleviating effect of two kinds of black tea aromas on physical and psychological stress induced by the Uchida-Kraepelin test, based on salivary chromogranin-A (CgA) levels as a stress marker and subjective evaluations (Profile of Mood States).

**Results:**

Compared with the water exposure control, inhaling black tea aroma (Darjeeling and Assam in this study) induced lower salivary CgA concentration levels after 30 min of mental stress load tasks. This anti-stress effect of black tea aroma did not differ between the two tea types even though the concentration of the anti-stress components in the Darjeeling tea aroma was higher than that in the Assam aroma. However, Darjeeling tea aroma tended to decrease the tension and/or anxiety score immediately after the first exposure.

**Conclusions:**

Inhaling black tea aroma may diminish stress levels caused by arithmetic mental stress tasks, and Darjeeling tea aroma tended to improve mood before mental stress load.

## Background

Tea is the second most frequently consumed beverage in the world next only to water. Tea consumption beneficially affects mood and attention, such as improving relaxation and concentration, reducing tiredness and psychological distress, increasing work performance, and so on [1–5]. Aroma has also been reported as one of the functional tea components affecting the central nervous system (CNS) and autonomic nervous system (ANS). Some of the tea aroma constituents, such as green odor and linalool, have been shown to have an anti-stress effect in animal and human studies [6–11]. Recently, green tea studies found that smelling green tea induces a positive emotion reflected by increased subjective ratings for relaxed feelings, elevated band power of alpha or/and beta in the frontal cortical region, and improved mental task performance [12, 13]. Jasmine tea aroma was reported to have sedative effects and activated the parasympathetic nerve [14, 15]. Yamashita et al. reported that flavored black tea, namely the Darjeeling tea combined with additive flavor (a mixture of passionfruit, grapefruit, and lavender oil) enhanced the relaxation of normal tea ingestion [16].

However, the scientific evidence of black tea aroma itself on the physiological response is still insufficient. There is no literature available comparing the anti-stress effects of different black tea aroma samples on ANS activities.

Chromogranin-A (CgA) is a major protein in the adrenal chromaffin cells and adrenergic neurons. As an ANS activity response to stress, CgA and catecholamines are co-released into the extra-cellular environment. Nakane found a prompt elevation in salivary CgA levels and a delayed increase in salivary cortisol levels when psychosomatic stress was induced through a test involving an oral presentation in front of an audience or a driving situation [17]. Nomura et al. also found that salivary CgA concentration showed an increase during the mental stress tasks and decreased (recovering) during the intermissions, demonstrating the possible candidacy of CgA as a biomarker for a short-term mental workload [18]. A study on green tea’s function showed that salivary CgA concentration levels increased after mental stress load tasks, while ingestion of green tea inhibited this increase and lowered the score on the Profile of Mood States (POMS), effects which both indicate the anti-stress effect of drinking green tea [4]. CgA was also reported in the evaluation of odor effects. Toda and Morimoto found that a lavender aroma inhalation lowered CgA levels which had been elevated by the arithmetic task [19]. These studies suggest that salivary CgA is a sensitive and promising index for psychosomatic stress.

In this study, we assessed the anti-stress effects of the two kinds of black tea aromas—Darjeeling and Assam—on CNS activities in healthy people by measuring salivary CgA. Warm water was used as a control sample. We also evaluated the POMS scores and the visual analogue scale (VAS) scores as subjective ratings of mental state.

## Methods

### Participants

Eighteen healthy volunteers (5 males, 13 females; age 20.4 ± 0.81 years) participated in three experimental trials from 10 to 11:30 am with an interval of 24 h between trials. All participants were requested to avoid eating or drinking anything but water for 3 h before the start of each trial. The experiment conducted in this study was approved by the research ethics committee of the Chubu University and was carried out in accordance with the Declaration of Helsinki.

### Procedure

**Fig. 1:**
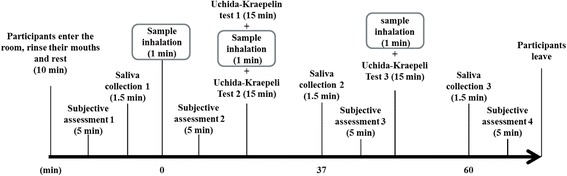
Procedures used in this study. Saliva collection and subjective assessments, including POMS and VAS, were performed before and again 30 and 45 min after the first inhalation of aroma sample. An additional subjective assessment was also completed immediately after the first inhalation

As shown in Fig. 1, all participants were required to complete sessions on a total of three study days in March. The room temperature was 25.8 °C. On the day of the experiment, after the participants entered the room, they were seated and asked to rest for 10 min. During the resting time, participants rinsed their mouths with a cup of water. After resting, the first subjective assessment (POMS) and the first saliva collection were completed to obtain baseline data (time 1). Participants then smelled one of the aroma samples for 1 min, followed by the second subjective assessment (time 2). They then began the first 15-min mental stress load task session (Uchida-Kraepelin (U-K) test 1). Immediately after the task, the second aroma inhalation (1 min) and the second stress load task (U-K test 2, 15 min) were performed. Subsequently, the second saliva collection and the third subjective assessment (time 3) were completed. Then the participants smelled the same aroma sample for the third time (1 min), again followed by the last 15-min stress load task (U-K test 3), third saliva collection, and finally the fourth subjective assessment (time 4).

### Treatment

A cross-over, single-blind, randomized design was used in this study. Three separate trials were performed in which the participants inhaled one of three beverage aroma samples (water as a control and two kinds of black tea aromas—Darjeeling and Assam) each day. Tea aroma samples were prepared by extracting 3 g of tea leaf with 180 ml 100 °C hot water for 5 min. After extraction, tea leaves were removed, and 100 ml of the tea sample was cooled to 60 °C and then poured into a brown vial bottle. Participants were asked to hold the bottle in a paper cup and open the cap for smelling. The warm water used as the control sample was prepared by cooling hot water to 60 °C. Fresh samples were prepared for each 1-min smelling session.

Levels of aroma components in the two tea samples were analyzed by solid-phase microextraction (SPME) and gas chromatography-mass spectrometry (GC-MS) method (AMR Inc., Japan and Thermo Electron Co., Ltd., Japan). The results are shown in Table 1.

**Table 1:** The levels of main functional components in the two black tea aroma samples (peak area divided by internal standard)

Compound name	Samples (IS ratio)	
	Darjeeling	Assam
Hexanal	0.5	0.2
E-2-hexenal	0.8	0.3
1-hexanol	0.3	ND
(Z)-3-hexen-1-ol	0.7	0.2
E-2-hexen-1-ol	0.3	ND
Linalool oxide (cis-franoid)	4.6	1.1
Linalool oxide (trans-franoid)	8.2	2.8
Linalool	13.9	5.2
Linalool oxide (cis-pyranoid)	0.8	0.5
Linalool oxide (trans-pyranoid)	1.8	ND
Nerol	0.4	ND
Geraniol	14.8	0.7
Phenylethyl alcohol	1.3	0.2

*ND* not detected

### Stress load task

The Uchida-Kraepelin (U-K) test, which was modified from Kraepelin’s arithmetic test and developed by Uchida [20], was used as the stress load task. This test has been widely used as a mental stressor in current literatures [21–23]. In this study, participants were given a pre-printed paper containing 15 lines of random, single-digit, horizontally aligned numbers and asked to perform calculations as quickly and accurately as possible for 15 min. Three sessions were performed in each trial. The average number of answers and percentage of correct answers for each test were used as indices of task performance.

### Saliva collection and CgA measurements

Saliva before the first sample inhalation was collected as the baseline data and collected after U-K test 2 and U-K test 3 at times of around 37 and 60 min from the first sample inhalation, respectively. Saliva was collected in Salivette tubes (Sarstedt, Germany) and centrifuged at 3000 rpm for 15 min at 4 °C. The supernatant was transferred into Eppendorf tubes and frozen at − 80 °C for later measurement. The concentration of CgA in the saliva samples was subsequently determined by enzyme-linked immunosorbent assay (Yanaihara Institute Inc., Japan). At the same time, total protein was measured with the dye-binding assay of Bradford (Bio-Rad Protein Assay, US). Measured values of CgA were divided by the protein concentration and used for further analysis.

### Subjective assessment

The VAS and POMS were used for subjective ratings on mood state. VAS was completed immediately after the first sample inhalation. POMS was performed before the first saliva collection for the baseline data and again after first sample inhalation and after the second and the last saliva collections.

Participants’ feelings of being under pressure, drowsiness, stress, relaxation, fatigue, ease of mind, tension, and four more questions asking how they felt about the aroma samples (preference, odor intensity, taste, and familiarity with the odor samples) were assessed using VAS. A continuous, 10-cm VAS rating scale was used, with the 0 end point representing “do not feel” and the 10 end point indicating “strongly feel”. Subjects were asked to make a mark on the scale at the point that represented their mood at the time immediately after the first sample inhalation.

We used a short version of POMS to assess distinct affective mood states. The POMS is a popular tool used widely among psychologists and scientists from many fields. Six identifiable mood or affective states were scored from the POMS: tension-anxiety, depression-dejection, anger-hostility, vigor-activity, fatigue-inertia, and confusion-bewilderment. Total Mood Disturbance (TMD) was then calculated from these six scores. A higher TMD score indicated a more negative affective state, that is, positive changes in mood were reflected by negative changes to TMD scores. All of the scores were used for analysis in this study.

### Determination of main tea aroma components

Table 1 shows the test results of selected main aroma components in the two black tea samples. Hexanal and hexanol were reported to attenuate mental stress response in rats [24–26]. Linalool had sedative effects on both autonomic nerve activity and mood states [14]. Geraniol, nerol, linalool, and phenylethyl alcohol, together with other main components of rose oil, induced inhibition of sympathetic activity [27].

The concentration of these components was approximately onefold or even higher in the Darjeeling tea than in the Assam. Thus, comparing the two tea aroma samples, we expected Darjeeling aroma would have a more anti-stress effect than Assam.

### Statistical analysis

Data were analyzed using IBM SPSS Statistics version 19. All data are expressed as the mean ± standard error, and *P* < 0.05 was considered significant.

VAS scores after the first odor inhalation and POMS, concentrations of CgA at each time point were analyzed by nonparametric Friedman tests to detect differences among the three odor samples (water, Darjeeling, and Assam). Time effects on POMS and CgA concentrations of each odor sample among the three time conditions (baseline, after U-K test 2, and after U-K test 3) were also detected by Friedman tests. Wilcoxon signed-rank tests with Bonferroni correction were then carried out for the comparisons between sample treatments and between time conditions.

Repeated measure one-way analysis of variance (ANOVA) and Bonferroni post hoc tests were performed to detect differences of task performance among the three sample conditions.

## Results

### VAS

**Fig. 2:**
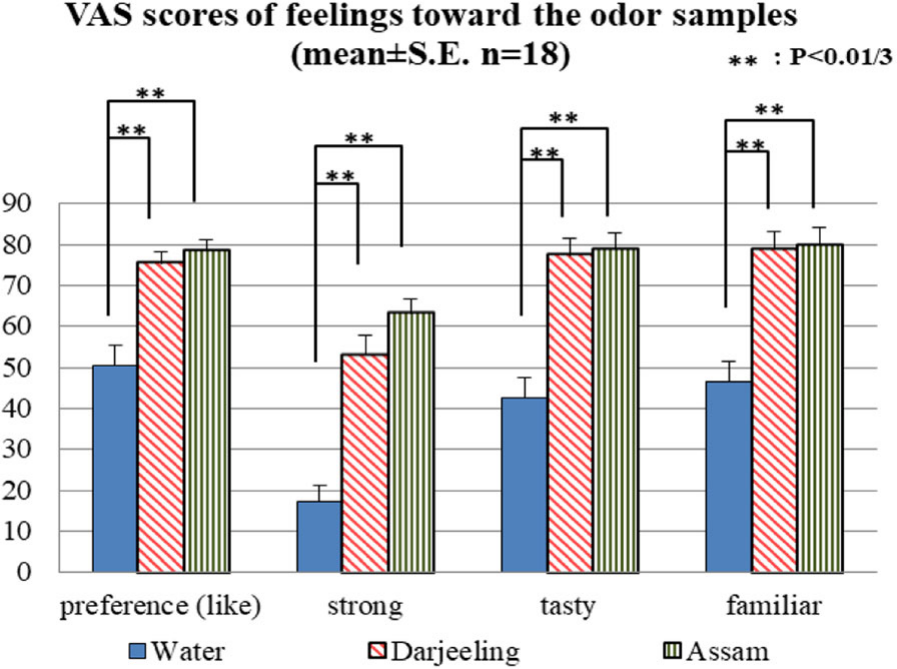
VAS scores of feelings regarding the aroma samples and the comparisons using Wilcoxon signed-rank tests with Bonferroni correction

VAS scores taken immediately after the first odor inhalation showed that feelings regarding the aroma samples (preference, odor intensity, taste, and familiarity with the aroma) were different among the three inhalation conditions, as measured by Friedman tests (*χ*^2^(2) = − 15.400, 28.000, 22.057, 14.111, *P* = 0.000, 0.000, 0.000, 0.001). As may be seen in Fig. 2, the results of Wilcoxon signed-rank tests with Bonferroni correction showed that the “like,” “strong,” “tasty,” and the “familiar” scores for water were significantly lower than those of the two tea aroma samples (*Z* = − 3.195, − 3.724, − 3.637, − 3.071 for Darjeeling and *Z* = − 3.376, − 3.724, − 3.637, − 3.223 for Assam; each *P* < 0.01/3).

### POMS

**Fig. 3:**
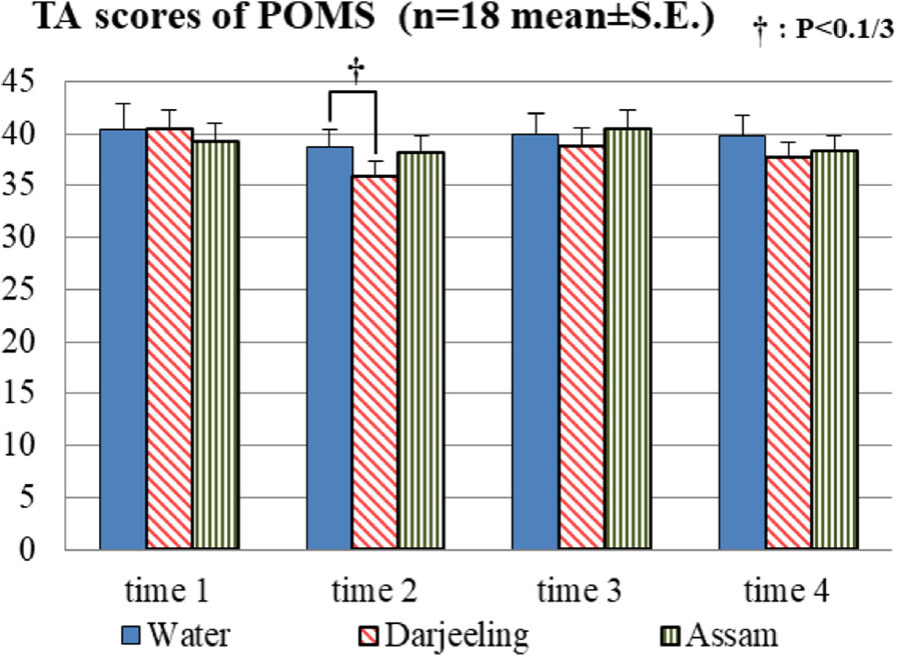
TA scores of POMS from each subjective assessment (time 1: baseline; time 2: immediately after the first inhalation; time 3: after U-K test 2; time 4: after U-K test 3) and the results of comparisons by Wilcoxon signed-rank tests with Bonferroni correction

The effect of aroma samples on the POMS scores as measured by Friedman tests showed differences only in tension and/or anxiety (TA) scores of time 2 among the three aroma treatment conditions (*χ*^2^(2) = 7.159, *P* = 0.028). Wilcoxon signed-rank tests indicated that the inhalation of Darjeeling tea aroma tended to decrease the TA score compared with the water condition (Fig. 3, *Z* = − 2.155, *P* < 0.1/3).

### CgA

**Fig. 4:**
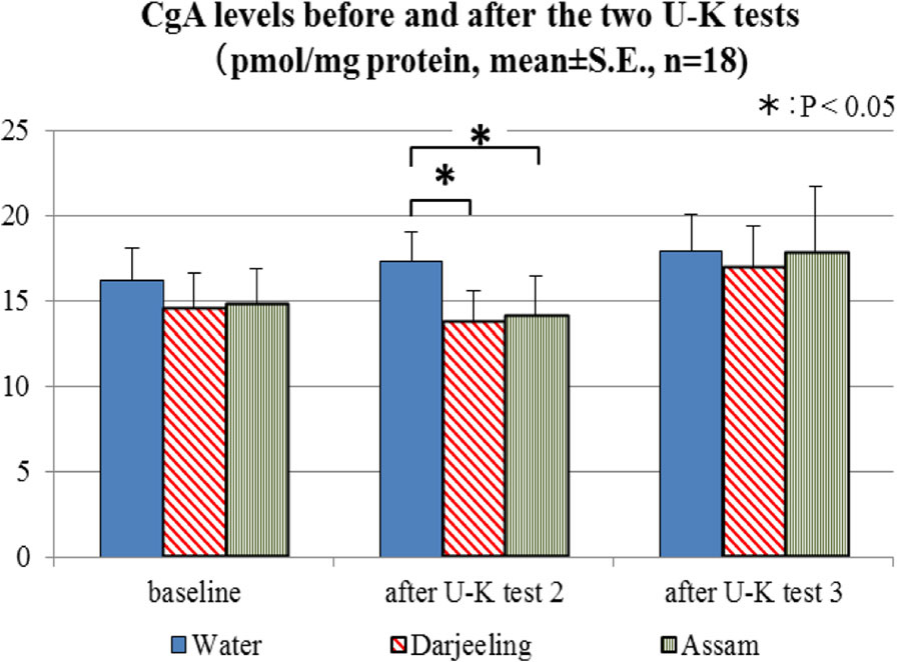
Salivary chromogranin-A (CgA) levels before and after U-K tests in each treatment condition. Comparisons were performed using Mann-Whitney *U* tests with Bonferroni correction

Among the three aroma treatment conditions, differences were only found immediately after U-K test 2 as measured by Friedman tests (*χ*^2^(2) = 10.111, *P* = 0.006). Wilcoxon signed-rank tests indicated that the CgA concentration after inhalation of water was higher than those in both the Darjeeling tea aroma condition and the Assam tea aroma condition (Fig. 4, *Z* = 2.543, 2.591; each *P* < 0.05/3). There was no difference among the three aroma samples at baseline measurements (*χ*^2^(2) = 1.333, *P* = 0.513).

### Task performance

There was no significant difference found in the arithmetic task performances among the three aroma conditions in this study.

## Discussion

We assumed that salivary CgA concentration levels are increased by mental stress load tasks [4, 19], and tea aroma samples may be able to reduce this increase due to the activities of their anti-stress components. Unexpectedly, the increases of CgA level by the U-K tests in this study were too small to elicit a significant statistical difference among the three time courses, i.e., baseline, after U-K test 2, and after U-K test 3. It could be that the U-K test did not work sufficiently enough as a mental stress load task for the participants recruited in this study. Various tasks may be needed to raise the stress level more prominently so as to elucidate the anti-stress effects more precisely and sensitively.

However, the two tea aroma treatments led to lower CgA levels than that in the water treatment after 30 min in total of the two stress load tasks (U-K test 1 and test 2). This indicated that inhaling black tea aroma can promote a less stressful state compared with the water sample. On the other hand, no difference between the two black tea aroma treatments could be found even though the concentration of the aroma components in Darjeeling tea aroma was much higher than that in the Assam aroma as shown in Table 1. It seems that the level of the anti-stress effect does not simply depend on the doses of these teas’ efficacious components.

Another factor which possibly influenced the mood during the assessments is the subjective feelings regarding the aroma samples. The “like,” “tasty,” and the “familiar” VAS scores for water were significantly lower than those for the two tea aroma samples. An odor study using green tea reported that green tea odor which had higher scores in “familiar” and “tasty” and a lower score in “dislike” caused a less anxious emotional state with a decline of right frontal cortical activity [13]. Thus, the positive feelings toward the two tea aroma samples in this study could have similarly contributed to the lower stress level after U-K tests indicated from the CgA results.

Finally, the results of POMS collected immediately after the first inhalation showed a trend for Darjeeling tea aroma to induce a lower TA score compared with water. Lower TA score represented a mood state of less tension and/or anxiety. On the other hand, Assam tea aroma failed to show any mood improving results in POMS scores. This suggested that the higher concentrations of the anti-stress components in Darjeeling tea aroma could have contributed to this stronger effect in promoting a more relaxed state than Assam tea aroma. Nevertheless, this effect attenuated with following repetitions of the aroma exposure. It is considered that a habituation of aroma responses may have occurred, and thus, the influence on psychological reaction diminished [28, 29]. This habituation may also be the reason why the concentration of CgA only changed among the odor sample conditions at the time after U-K test 2 but no differences after U-K test 3. That is to say, the odor effect may only exist for a short time and only before the stimulus being accustomed to.

One of the limitations of our study is that only the concentration of the salivary CgA was used as the index for the physiological activity. Another factor to consider for future studies could be to explore gender differences in the anti-stress effects of black tea aroma, for which more male participants will be needed. It will also be interesting to compare this data with other kinds of tea odors by multiple indexes of ANS and/or CNA activities.

## Conclusions

Inhaling black tea aroma induced lower salivary CgA concentration levels after 30 min of mental stress load tasks compared with water odor condition. This anti-stress effect of the black tea aroma did not differ between the two types of tea even though the concentration of the anti-stress components in Darjeeling tea aroma was higher than in Assam aroma. On the other hand, Darjeeling tea aroma decreased the TA score (POMS) immediately after the first exposure. The above results indicated that inhaling black tea aroma may diminish stress levels caused by arithmetic mental stress task, and Darjeeling tea aroma tended to improve mood before mental stress load. At the same time, our results also suggested that the physiological and the psychological responses to odor stimuli may not be always consistent simply to the doses of the functional components when focus on anti-stress effects. More studies on elucidating the mechanism of anti-stress response are needed to help improve human adaptability to the current stressful social life and to create a healthy and comfortable living environment.

## Abbreviations

ANOVA: Analysis of variance
ANS: Autonomic nervous system
CgA: Chromogranin-A
CNS: Central nervous system
POMS: Profile of Mood States
TA: Tension and/or anxiety
U-K: Uchida-Kraepelin
